# Function and Pharmacology of Spinally-Projecting Sympathetic Pre-Autonomic Neurones in the Paraventricular Nucleus of the Hypothalamus

**DOI:** 10.2174/157015911795596531

**Published:** 2011-06

**Authors:** Nicolas Nunn, Matthew Womack, Caroline Dart, Richard Barrett-Jolley

**Affiliations:** Centre for Integrative Mammalian Biology, University of Liverpool, Brownlow Hill & Crown St. Liverpool, L69 7ZJ, UK

**Keywords:** Blood pressure, penile erection, GABA, PVN, paraventricular nucleus, hypothalamus, cardiovascular, sympathetic, parvocellular mediocellular, neuropeptides, oxytocin, substance P, tachykinin, vasopressin, angiotensin, pharmacology.

## Abstract

The paraventricular nucleus (PVN) of the hypothalamus has been described as the "autonomic master controller". It co-ordinates critical physiological responses through control of the hypothalamic-pituitary-adrenal (HPA)-axis, and by modulation of the sympathetic and parasympathetic branches of the central nervous system. The PVN comprises several anatomical subdivisions, including the parvocellular/ mediocellular subdivision, which contains neurones projecting to the medulla and spinal cord. Consensus indicates that output from spinally-projecting sympathetic pre-autonomic neurones (SPANs) increases blood pressure and heart rate, and dysfunction of these neurones has been directly linked to elevated sympathetic activity during heart failure. The influence of spinally-projecting SPANs on cardiovascular function high-lights their potential as targets for future therapeutic drug development. Recent studies have demonstrated pharmacological control of these spinally-projecting SPANs with glutamate, GABA, nitric oxide, neuroactive steroids and a number of neuropeptides (including angiotensin, substance P, and corticotrophin-releasing factor). The underlying mechanism of control appears to be a state of tonic inhibition by GABA, which is then strengthened or relieved by the action of other modulators. The physiological function of spinally-projecting SPANs has been subject to some debate, and they may be involved in physiological stress responses, blood volume regulation, glucose regulation, thermoregulation and/or circadian rhythms. This review describes the pharmacology of PVN spinally-projecting SPANs and discusses their likely roles in cardiovascular control.

## 1. INTRODUCTION

**Fig. (1):**
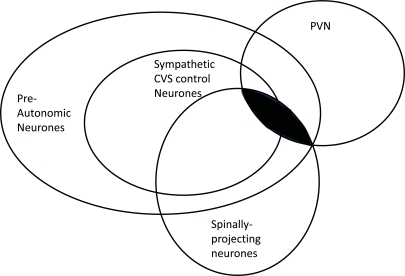
**Spinally-projecting sympathetic pre-autonomic neurones of the paraventricular nucleus of the hypothalamus (PVN)**. The PVN consists of several sub-populations of neurones some of which project to the spinal cord. There are likely to be each combination indicated in the Venn diagram. This review focuses on the shaded area; spinally-projecting sympathetic pre-autonomic neurones (spinallyprojecting SPANs) of the PVN. Many of these target the cardiovascular system (CVS).

The paraventricular nucleus (PVN) of the hypothalamus is a critical regulator of numerous endocrine and autonomic functions [1-5]; Loewy [6] referred to it as being the “autonomic master controller”. Many of these autonomic functions are served by spinally-projecting neurones (Fig. **1**), a number of which have been shown to be involved in cardiovascular regulation [7]. These and other spinally-projecting sympathetic pre-autonomic neurones (SPANs) in the PVN are therefore attractive targets for pharmacological manipulation of both the cardiovascular system and potentially, other autonomic systems.

**Fig. (2):**
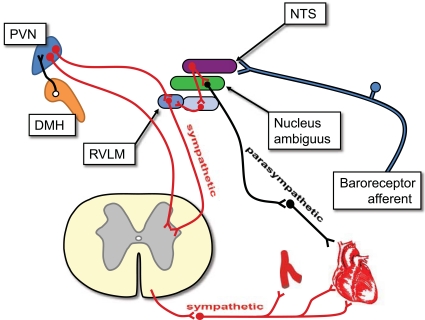
**Central cardiovascular control in the rat**. The cardiovascular system is largely controlled by areas of the brainstem; however, there is also strong evidence that centres in the forebrain, including the PVN, exert “top-level” control via both medulla-projecting and spinally-projecting SPANs. Afferents from carotid baroreceptors ascend via cranial nerve IX (the glossopharangeal nerve), while afferents from the aortic bodies ascend *via* cranial nerve X (the vagus nerve). These afferents are processed by the nucleus of the solitary tract (NTS). Parasympathetic drive emanates from the nucleus ambiguus, whilst sympathetic drive emanates from a number of locations, in particular the rostroventrolateral medulla. The role of the medulla in cardiovascular control is reviewed by [9].

The cardiovascular system is subject to numerous levels of control. Although the basic functions of the heart and blood vessels are controlled locally, the whole system is centrally regulated by the brain [8, 9]. Part of the central regulation comes from the medulla, which allows rapid adaptation to changing homeostatic challenges. Anticipatory regulation, however, allows for an increase in cardiac output just prior to increased systemic oxygen demand. This was termed the “preparatory reflex” [10] and appears to be the highest level of cardiovascular control. The process is largely attributed to an increase in sympathetic activity, and was first identified by Walter Cannon [11]. This reflex is now more commonly referred to as the “fight-or-flight” response, the “stress response”, or the “defence reaction”. Inappropriate activation of this system may lead to hypertension and could account for the increased cardiac death resulting from stressors such as exercise [12], anger [13] or emotional shock [14]. Furthermore, some aspects of this pathway appear to be activated in chronic heart failure [15]. Forebrain regions involved in cardiovascular regulation were discovered early in the 20^th^ century by stimulation or ablation of specific areas [16, 17]. Later, several areas of the hypothalamus (Fig. **2**) were shown to be of particular importance [e.g.,^ 18^,^ 19^,^ 20^]. These include areas we now know as the PVN and the dorsomedial hypothalamus (DMH), although both were poorly defined at the time. The PVN comprises approximately 21,500 neurones, approximately three quarters of which make up the parvocellular subdivision [21], which includes both spinally-projecting SPANs and hypothalamic-pituitary-adrenal (HPA) axis control neurones [3, 6, 22]. 

Estimates suggest that up to 2000 neurones project directly from the PVN to the intermediolateralis (IML) of the spinal cord [23-25], and terminate close to sympathetic pre-ganglionic neurones [26, 27] and are, in this review, collectively termed spinally-projecting sympathetic pre-autonomic neurones (spinally-projecting SPANs). SPAN cell bodies are found most densely in a region of the PVN termed the parvocellular subnucleus, particularly in a subdivision referred to by some authors as the mediocellular region [21]. 

**Fig. (3):**
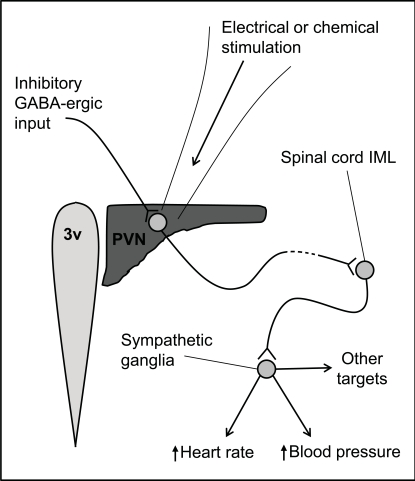
**Actions of spinally-projecting SPANs originating in the paraventricular nucleus (PVN) of the hypothalamus.** The PVN lies alongside the 3^rd^ ventricle (3v), and contains a number of neurones which project to the intermediolateralis (IML) of the spinal cord. When stimulated with excitatory neurotransmitters or electrical current, they activate sympathetic pre-ganglionic neurones in the IML which, in turn, increase heart rate and blood pressure and glucose secretion. They may also increase TSH release. These PVN neurones are tonically inhibited by GABA input. Please see main text for references.

Spinally-projecting SPANs are strongly implicated in cardiovascular control [7, 28-30], but their primary role is unknown (Fig. **3**). Several hypotheses have been proposed such as; circadian regulation of blood pressure [31], blood-volume regulation [24, 25] and the cardiovascular response to psychological stress [32]. Furthermore, elevated sympathetic activity is associated with congestive heart failure [15, 33, 34], and may also be associated with diminished GABA sensitivity of PVN neurones [15, 35, 36]. This review will discuss the neuropharmacology and function of spinally-projecting SPANs of the PVN. In the majority of cases these experiments have focussed on control of the rat cardiovascular system, although recently, study of spinally-projecting SPANs has broadened to entirely new systems such as glucose regulation, thermoregulation and penile erection.

## 2. ELECTROPHYSIOLOGY OF SPINALLY-PROJECTING SPANS

A number of *in vitro* studies have investigated the electrophysiological properties of PVN neurones [37-40]. They show that parvocellular neurones (termed “PVN type II” neurones) express a slowly inactivating delayed rectifier potassium conductance. Conversely, the neurosecretory magnocellular neurones of the PVN (termed “PVN type I” neurones) appear to express a rapidly inactivating (A-type) potassium conductance. Fewer studies have been conducted on *identified* spinally-projecting SPANs; medulla-projecting neurones show strong inward rectification and “A-type” potassium conductance [41, 42] and spinally-projecting SPANs show a slowly inactivating potassium conductance [43]. More recent studies have also identified ATP dependent potassium channels [44, 45], which may serve to couple glucose levels to sympathetic activity. Pharmacological characterisation of the potassium channels involved is possible using potassium channels inhibitors [46-53], although confirmation requires immunohistochemical or RT-PCR approaches since most of these inhibitors lack high selectivity. When recorded from *in vitro* most spinally-projecting SPANs fire action potentials spontaneously [41, 54], but they are apparently quiescent* in vivo* [55-58]. This implies that the tonic inhibition of spinally-projecting SPANs may be, in part at least, lost in the preparation of brain-slices for *in vitro* recording.

## 3. NEUROTRANSMITTERS RELEASED BY SPANS

Discussion of the neuropharmacology of SPANs can include neurotransmitters released by the neurones and neurotransmitters acting upon them. The first of these questions has been approached by the use of retrograde/anterograde labelling, trans-synaptic tracing, immunohistochemistry and *in situ *hybridisation. Using these techniques it has been demonstrated that spinally-projecting SPANs express met-endorphin (20%), dynorphin (up to 40%), oxytocin (up to 40%) and vasopressin (up to 40%) [59-61]. However, a significant proportion also produce dopamine, met-enkephalin (10%), leu-enkephanlin, somatostatin, angiotensin II and atrial natriuretic peptide [62-65]. This clearly implies that some SPANs express more than one neuropeptide neurotransmitter. Interesting questions which remain unanswered include: what combinations of neurotransmitters are expressed by particular neurones, and, do different neurotransmitters serve different functions? For example, the closely related neuropeptides oxytocin and vasopressin are commonly co-expressed [see for example 66]. 

The anatomical evidence that vasopressin acts a SPAN neurotransmitter is supported by functional experiments in which PVN neurones were stimulated, producing an immediate increase in renal sympathetic nerve activity (rSNA) and mean arterial blood pressure [67]. However, these effects were prevented by lower thoracic intrathecal preloading with the vasopressin 1a (V_1a_)-receptor antagonist *d*(CH_2_)_5 _ [Tyr(me)^2^,arg^8^vasopressin [67]. Interestingly, since oxytocin exerts effects both through the oxytocin receptor (OT-R) and through the V_1a_–R [68], some of the effects attributed to oxytocin may in fact be mediated by the latter. However, oxytocin is clearly important as a neurotransmitter of SPANs, since both vasopressin and oxytocin levels increase in the spinal cord when the PVN is stimulated [69]. Additionally, the tachycardia associated with PVN stimulation is reduced by the highly selective OT-R antagonists d(CH_2_)_5_[Tyr(Me)^2^, Orn^8^]-oxytocin and L-368,899 [70]. One explanation suggested for the apparent disparity of the effects of oxytocin and vasopressin antagonists is that SPAN neurotransmission in the upper thoracic spinal cord is mediated *via *oxytocin, but in the lower thoracic cord it is mediated by vasopressin [30]. 

Dopamine has also been strongly implicated as a SPAN neurotransmitter [65], but its activity is complex. Several reports demonstrate that dopamine, and its mimetic apomorphine, excite sympathetic pre-ganglionic neurones in rats (ie. the neurones that SPANs would act upon) [71-74]. However, evidence also suggests that it may serve as an inhibitor of sympathetic activity [75]. Data supporting the function of the other putative SPAN neurotransmitters described above is, so far, more disparate. For example, intrathecal glutamate antagonists reduce the increases in rSNA seen following chemical stimulation of the PVN [75] suggesting that the excitatory amino acid glutamate may act as a SPAN output neurotransmitter. However, whilst enkephalins and angiotensin II are both produced by SPANs [59, 60, 62-64] and modulate sympathetic pre-ganglionic neurones [76-78] in rats, pharmacological block of their respective receptors (at the level of the spinal cord) has not been shown directly to prevent the sympathetic effects of PVN stimulation. This may be because SPANs expressing these neurotransmitters innervate pre-ganglionic neurones targeting less well characterised sympathetic effectors in the viscera [79] or simply that appropriate experiments to detect such effects have yet to be conducted. The amino acid γ-aminobutyric acid (GABA) is the most abundant inhibitory neurotransmitter in the central nervous system [80]. Recent analysis of both retrograde and trans-neuronal labelling data shows that whilst spinally-projecting SPANs receive GABA-ergic synapses, they do not contain GABA themselves [81]. 

In addition to the pathways leading from the PVN to the IML thence to the heart and blood vessels there are pathways projecting from the superchiasmatic nucleus (SCN), to the thyroid and to the liver, *via *the PVN [82-84]. Many neurones in this pathway express thyroid stimulating hormone (TSH) [82], possibly in the PVN spinally-projecting SPANs themselves.

## 4. NEUROTRANSMITTERS ACTING ON SPANS

**Fig. (4):**
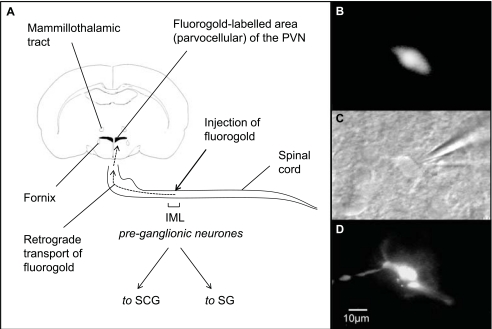
**Methods for patch-clamping retrogradely-labelled neurones. A,** the retrograde tracer fluorogold is injected into the rat intermediolateralis (IML) at level T2-T4, it is also possible to use other tracers, such as rhodamine-labelled microspheres (see Fig. 8). The IML is dense with pre-ganglionic neurones that project to the superiocervical (SCG) and stellate (SG) ganglia, and from there to the heart and blood vessels [65, 227]. The appearance of a fluorogold-labelled neurone **B**, prior to patch clamp recording, **C**, during patch-clamp, under near infrared differential interference contrast microscopy, and **D**, when patched with Lucifer yellow (a fluorescent dye) in the patch clamp pipette. The dye fills the neurone, and this gives re-confirmation that recording was from the appropriate cell. Reproduced from [43], with permission.

Far more studies have been published looking at the neurotransmitters expressed by SPANs than looking at neurotransmitters *acting on* SPANs. This is disappointing since, arguably, knowledge of the receptors expressed by a cell gives greater therapeutic potential than knowledge of the transmitters released by it. A useful approach has been the combination of retrograde labelling and patch-clamp recording. In these studies, tracer is injected into the IML of the spinal cord, and a few days later brain slices are prepared. Spinally-projecting neurones are then clearly visible and can be targeted for electrophysiological study (Fig. **4**).

### 4.1. Amino Acid Neurotransmitters

A number of *in vivo* studies have investigated the neurotransmitters acting upon SPANs, or the receptors expressed by them, but there have been few *in vitro *studies on identified spinally-projecting SPANs. *In vivo* electrophysiological studies on cats confirmed an anticipated monosynaptic connection between the PVN and the spinal sympathetic motor area (the IML) [85]. Furthermore, electrical or chemical stimulation of the PVN was shown to generate a rapid rise of blood pressure and rSNA in conscious rats [86]. Further *in vivo *studies showed that PVN neurones receive tonic GABA-ergic inhibition, which maintains their spontaneous action potential discharge at relatively low rates [55, 57, 58, 87], despite additional excitatory glutaminergic input [86]. 

There is accumulating evidence that “tonic” inhibition is often mediated by GABA arising from extra-synaptic sources; sometimes referred to as “volume” GABA neurotransmission [88-89]. This correlates with the expression of α_5_-, or α_6_-type, and δ-type GABA_A_ receptor subunits [90, 91]. However, this has not yet been specifically examined with spinally-projecting SPANs. It may be an important issue to explore though, since a unique pharmacological profile for extra-synaptic GABA receptors is emerging, [91-93] which may become therapeutically exploitable. 

In the case of SPANs, loss of tonic inhibition would lead to an increase in sympathetic outflow. Excessive sympathetic outflow could potentially be reduced by selective activators of “volume” GABA receptors. Furthermore, the degree of tonic inhibition of sympathetic outflow is, in part at least, dependent upon the local uptake of GABA by transporter proteins, in particular GAT3 located on glial cells [94]. Therefore, there are a number of means by which parvocellular GABA inhibition could be targeted with the aim of reducing sympathetic outflow and blood pressure. 

Excitation of the PVN by either glutamate or the GABA_A_ receptor antagonist bicuculline increases blood pressure and heart rate, due to the tonic silence of SPANs [55, 56, 86, 87, 95]. This has led to many* in vitro* studies investigating control of SPANs to focus on the role of GABA. In fact, a number of studies have demonstrated the presence of GABA_A_ receptor currents in the PVN [38, 87, 96-100]. The parvocellular region of the PVN, which contains the majority of SPANs, expresses a high density of GABA_A_ α_2_ –subunits [101]; this was also seen in retrogradely labelled spinally-projecting SPANs [102]. Additional studies have shown spinally-projecting parvocellular neurones to be inhibited by GABA [31, 54, 103-104], as predicted by the earlier *in vivo* work. Interpretation of both the *in vivo* and *in vitro* work is further complicated by some frequently overlooked variables. Firstly, there is little consideration of the role of pre-synaptic GABA receptors. These are typically of the GABA_B_ receptor subtype and have been shown to inhibit both inhibitory and excitatory input to spinally-projecting SPANs [105]. Secondly, the primary GABA_A_ antagonist used to investigate possible GABA involvement is bicuculline or bicuculline methiodide, both of which are potassium channel blockers [106-108]. The effect of potassium channel inhibition would appear very similar to block of GABA_A_ receptors; excitation. 

Neuroactive steroids and nitric oxide (NO) are well known modulators of GABA_A_ receptors. In the PVN, for example, tetrahydrodeoxycorticosterone (THDOC) [103] modulates spinally-projecting SPANs and NO modulates rostral ventrolateral medulla (RVLM)-projecting SPANs [109]. Both effects are mediated by modulation of GABA_A_ receptors [110]. However, other steroid hormone receptors, such as oestrogen β-receptors [111], are expressed by SPANs, which raises the possibility that some steroids may modulate sympathetic outflow by this mechanism. Other mechanisms may also be involved, for example cortisol is known to activate SPANs at high doses *via *an inhibition of potassium channels [100].

SPANs are excited by the excitatory amino acid neurotransmitter glutamate [31], mediated by the activation of at least two fast ionotropic glutamate conductances. This is carried largely by α-amino-3-hydroxy-5-methyl-4-isoxazole-propionic acid (AMPA) receptors (i.e., sensitive to 2,3-dihydroxy-6-nitro-7-sulfamoyl-benzo[f]quinoxaline-2,3-dione, NBQX, an AMPA receptor antagonist), but with an additional component of N-methyl D-aspartate (NMDA) conductance (i.e., sensitive to the classical NMDA antagonist amino-5-phosphonovaleric acid, AP-5, [31]). 

Combined electrophysiological and microdialysis studies on RVLM-projecting PVN neurones have shown that at least some of their glutaminergic input is also tonically inhibited by GABA_A_-receptors. Since glutaminergic neurotransmission is ubiquitous within the central nervous system, there has been particular recent focus on non-glutaminergic excitation of SPANs. This is because antagonism of the relatively sparse neuropeptide receptors could become therapeutically useful when action of amino acid receptor antagonists proves to be too widespread. 

### 4.2. Neuropeptide Neurotransmitters

Leptin [112], angiotensin II (AGII) [113, 114], neuropeptide W [115] and each of the endogenous tachykinins [54] have all been investigated as pharmacological regulators of spinally-projecting SPANs. The strongest candidates appear to be AGII and the tachykinin substance P (SP), with both neuropeptides acting indirectly *via *GABA neurotransmission [54, 114]. 

#### 4.2.1. Angiotensin II

The neuropeptide AGII, expressed by PVN neurones [116], acts at two principal receptors: AT_1_ and AT_2_. AT_1_ is the more abundant of the two [117-118] and, in rodents, is encoded by two genes: AT_1A_ and AT_1B_ [119]. However, it is not clear whether both of these subtypes exist in humans, nor which subtype is more important for cardiovascular control in rodents. It has been suggested that AT_1B _is more important for control of blood pressure in mice [120], but *in situ* hybridisation studies show that AT_1A_ is expressed more richly in the parvocellular region of the rat PVN [121], the region particularly important for autonomic control. Integrative experiments showed that AGII injection into the PVN gave an AT_1_-dependent increase in blood pressure [122], and single-unit recording studies of antidromically-identified neurones demonstrated that this involved spinally-projecting SPANs [123]. Later, the same group demonstrated inhibition of A-type potassium currents by AGII, acting *via *an AT_1_ receptor [124]. Whilst AGII directly modulates medulla-projecting PVN neurones [125] to elevate blood pressure, spinally-projecting SPANs do not express AT_1_ receptors [113]. Therefore, this implies that AGII activates SPANs indirectly, and this was investigated using whole-cell recording from retrogradely labelled spinally-projecting SPANs [114]. In these experiments, AT_1_ receptors were found to co-localise with synaptophysin (a pre-synaptic marker protein). In addition, AGII decreased the frequency and amplitude of spontaneous inhibitory post-synaptic potentials in SPANs. This strongly suggests that AGII excites spinally-projecting SPANs by reducing the tonic GABA-ergic input. 

#### 4.2.2. Tachykinins

**Fig. (5):**
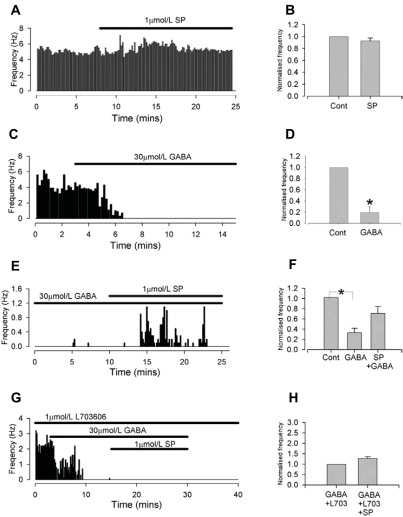
**Substance P (SP) increases the rate of action potential discharge of individual neurones in brain slices in the presence of GABA.** Brain slices were taken from adult rats, and action potentials recorded from single neurones in the PVN. **A**, representative results showing SP applied to SPANs under control conditions; **B**, mean results, showing SP alone has no effect on rate of action potential discharge. **C**, representative results showing application of GABA; **D**, mean results showing that GABA inhibits spontaneous action potential frequency. **E**, representative results showing SP added during GABA inhibition; **F**, mean results showing that SP increases the rate of action potential discharge against a background of tonic GABA inhibition. **G**, Representative results showing SP interaction with NK1 antagonism; **H**, mean results showing the effects of SP are abolished by the selective NK1 antagonist L703606. Cont = control. Reproduced from [54], with permission.

**Fig. (6):**
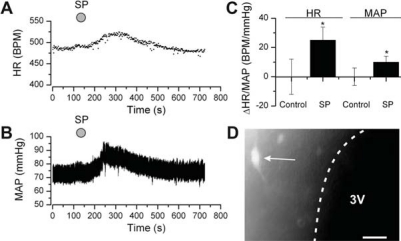
**Substance P (SP) injected into the PVN elicits a rise in heart rate and blood pressure.** Heart rate and blood pressure were recorded in anaesthetised adult rats. Representative records of **A**, heart rate and **B**, blood pressure during intra-PVN injection of SP/FITC-SP (solution of SP and FITC-conjugated SP). **C**, mean data from a number of experiments. **D**, epifluorescent image of the medial PVN, after SP/FITC-SP intra-PVN injection. “3V” indicates the third ventricle; the white arrow indicates a parvocellular neurone labelled with FITCSP. Scale bar is 50µm. “SP” indicates SP/FITC-SP injection. Reproduced from [54], with permission.

The tachykinin family of neuropeptides, which includes substance P (SP), neurokinin A (NKA) and neurokinin B (NKB), is involved in central cardiovascular control [126]. Studies have shown high levels of tachykinin receptor expression within the PVN [127-129] and in our own studies we showed that injection of SP into the PVN increases heart rate, blood pressure (Fig. **6**) and rSNA [54]. These effects correlated with our observation of a strong inhibition of SPAN GABA_A_ receptors by SP [54]. Activation of SPANs by SP in brain slice experiments was only apparent when neurones were first inhibited by GABA (Fig. **5**). 

**Fig. (7):**
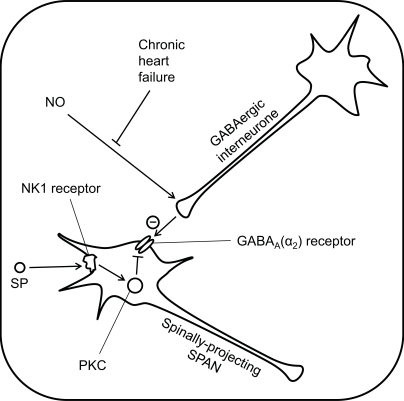
**Pharmacological control of SPANs within the PVN.** Spinally-projecting SPANs are tonically inhibited by GABA input, which is normally enhanced by nitric oxide (NO). However, during chronic heart failure, this potentiation of GABA is lost, leading to an overall increase in sympathetic activity. Substance P (SP) causes indirect activation of spinally-projecting SPANs by inhibition of the inhibitory GABAA receptors, *via* a protein kinase C (PKC)-dependent pathway.

All three of the principal mammalian tachykinin receptors (NK1, NK2 and NK3) are 7-transmembrane G-protein coupled receptors, although they act through a range of intracellular pathways, typically *via *pertussis toxin independent G-proteins [130, 131]. Of particular interest is that NK1 receptors activate protein kinase C (PKC) [131], and that PKC-dependent phosphorylation of GABA_A_ can inhibit GABA_A_ currents [132]. This was first shown using cultured cells expressing recombinant GABA_A_ subunits. However, SPAN activation by SP in the PVN was also found to be both PKC and NK1 receptor dependent (Figs. **5** and **7**) [54]. No effects of NKA or NKB agonists were observed (NKA4-10 and senktide, respectively). An interesting question remains; from where do the tachykinin inputs to SPANs arise? Of note, several intra-hypothalamic tachykinin pathways terminate in the PVN [133]. Furthermore, in brain slice experiments we found that SPAN activation following glutamate stimulation of the DMH was inhibited by tachykinin receptor antagonists [134]. This may prove significant since there is evidence that both the DMH and the PVN are important in the control of the cardiovascular system [135].

#### 4.2.3. Vasoctive Intestinal Peptide (VIP) and Pituitary Adenylcyclase Activating Peptide (PACAP) 

Retrograde labelling evidence has demonstrated the presence of vasoctive intestinal peptide/pituitary adenylcyclase activating peptide receptors type 2 (VPAC_2_) on spinally-projecting SPANs [136]. *PACAP*_(1 to 38)_ induced *c-fos *expression in PVN spinally-projecting neurones and this was abolished by a VPAC_2_ receptor antagonist [136]. It seems likely, from functional and viral tracing studies that these neurones are also activated by thyroid hormone (T3) [137], although receptors have not yet been identified specifically on the PVN spinally-projecting SPANs. Both the VPAC_2_ receptors and T3 nuclear receptors (TR) appear to be involved with control of endogenous glucose production by the liver, see below for further discussion [136, 137].

### 4.3. Other Receptors on Spinally-Projecting SPANs 

Purinergic receptors were originally classed as either P1 or P2 subtypes [138]. The P1 receptors are now termed adenosine receptors, A_x_ and are seven transmembrane G-protein couple receptors [139]. The P2 receptors are the ligand-gate cation channels activated by nucleotides [139]. Examples of both families of receptors have now been identified on PVN SPANs [45, 140], but only P1 on identified spinally-projecting SPANs [45]. Adenosine is inhibitory to several types of muscle cell, by acting *via *A_1_ receptors and K_ATP_ channels [141-143]. Li *et al.* [45] has now established that adenosine acts on spinally-projecting SPANs in a similar manner. P2X receptors have been identified on RLVM-projecting SPANs [140], but it remains to be seen if these receptors are also expressed on spinally-projecting SPANs. There are little data supporting the presence of other receptors on spinally-projecting SPANs. Price *et al.* [115] used multiplex single-cell RT-PCR experiments in combination with whole-cell recording in brain slices to show inhibition of SPAN neurones by neuropeptide W. This study did not use retrograde labelling to identify SPANs, but rather electrophysiological “finger-printing” and post-hoc identification of neuronal expression of vasopressin or oxytocin. Neuropeptide W is thought of as being a regulator of feeding behaviour (see for example [144]) and in this case was found to be acting through the neuropeptide W type 1 (NPBW1) receptor. 

**Fig. (8):**
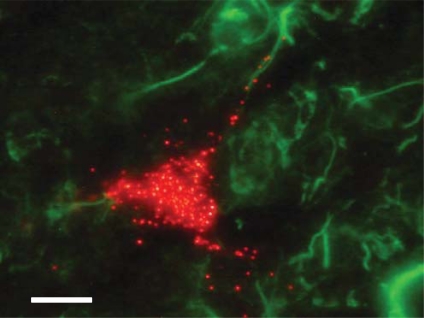
**Spinally-projecting SPANs do not co-localise with leptin receptor (OB-R) in rat PVN.** Spinally-projecting SPANs were labelled with rhodamine beads (red) using retrograde labelling (see Fig. 2) in an adult rat. A 40µm cryo-slice of the PVN was counter-stained for OB-R (green), using primary goat anti-OB-R IgG, Santa Cruz biotechnology; secondary rabbit anti-goat, Jackson. Scale bar is 10µm. Unpublished data.

Receptors for neuropeptide FF are densely expressed in the PVN and excite both magnocellular [145] and parvocellular [146] PVN neurones. These include neurones that project to the nucleus of the solitary tract [147], but there has been no investigation of their effects specifically on spinally-projecting SPANs. Evidence also suggests that leptin [148-150] may influence SPANs of the PVN. The leptin receptor (OB-R) is highly expressed in the PVN [151], and PVN neurones are depolarised by leptin [152]. However, leptin does not increase *c-fos* expression in spinally-projecting SPANs [112], suggesting that the PVN neurones being activated by leptin are not SPANs. Indeed, our own immunohisto-chemical data show that OB-R is expressed by neurones immediately surrounding SPANs (unpublished data, (Fig. **8**)), but not by the spinally-projecting SPANs themselves. Orexin has also been shown to depolarise neurones within the PVN, but again, this has not been shown specifically with SPANs [153]. It has been suggested that other neurotransmitters such as adrenomedullin [154] may also activate spinally-projecting SPANs, however, this has not yet been shown directly. 

## 5. PHYSIOLOGICAL IMPLICATIONS OF SPAN ACTIVITY

Spinally-projecting SPANs of the PVN influence the cardiovascular system [7, 155] (Fig. **3**), and there is clear evidence that the functioning of these neurones is altered during heart failure [15, 35]. However, the role of these neurones in normal physiology remains controversial. There is evidence PVN SPANs are involved in physiological functions as diverse as stress, thermoregulation and penile erection. 

### 5.1. Involvement with Stress Response

In addition to the clear increases in blood pressure observed with physical activity [156], the simple act of anticipating exercise [157] or performing mental arithmetic [158-159] is sufficient to produce a cardiovascular response. In excess, this preparatory reflex leads to increases in heart rate and blood pressure of a pathological nature [160-162]. Duan *et al.* showed that the stress “phenotype” could be reproduced by stimulation of the PVN [163] and non-specific chemical inhibition of the PVN prevents it [164]. Certainly, the PVN is central to the integration of the hormonal response to stress [5] and it also well known that stimulation of SPANs in the PVN exerts a powerful influence on blood pressure and heart rate and sympathetic nerve activity [55-57, 86, 95, 165-167]. 

*In vivo* experiments suggest that tachykinins play a major role in cardiovascular control and the expression of a cardiovascular stress response [126]. For example, SP is found at high levels within the PVN [168], and central tachykinins increase efferent sympathetic nerve activity [54, 169]. Furthermore, injection of tachykinin agonists into the hypothalamus [126, 170], and specifically the PVN [54, 171, 172], leads to rapid elevation of heart rate and blood pressure (Fig. **6**). Crucially, central administration of tachykinin antagonists reduces the elevation of blood pressure and heart rate resulting from psychological stress of conscious rats [173]. Trans-synaptic retrograde labelling experiments show a direct coupling between the stellate ganglion (which supplies sympathetic motor neurones to the heart) and the PVN [32], implicating PVN SPANs in control of the stress response. In fact PVN SPANs were included in the set of “central command neurones” identified by Jansen* et al.* [32], and inferred from the work of Walter B. Cannon in the early 1900s [174]. 

However, some scientists now reject this idea; several lines of evidence suggest that whilst the PVN is activated by stress, and stimulation of the PVN elicits increases in blood pressure and heart rate, it is the DMH which is important for the cardiovascular response to stress [135]. This conjecture is based on the observation that injection of the GABA_A_ antagonist bicuculline into the DMH prevents the cardiovascular response to air-jet stress, whereas injection into the PVN does not [175]. Whether this PVN SPAN-independent cardiovascular response to stress is general, or stress paradigm-specific, remains to be seen. Furthermore, since the activation of SPANs by SP involves the relief of GABA_A_ inhibition [54], inhibition of the PVN by GABA may not be the ideal means of testing this hypothesis. Non-specific PVN lesion experiments such as Busnardo and colleagues’ recent work [164], or specific block of spinally-projecting SPAN neurotransmitter receptors may resolve the controversy.

### 5.2. Control of Blood Volume

It has been suggested that spinally-projecting PVN neurones may be involved in the response to changes in blood volume [24, 176, 177]. This can, to a certain extent, be differentially regulated from blood pressure, by the cardiovascular system. The atrial/venous side of the cardiovascular system is relatively compliant and “expands” to accommodate increased blood volume with relatively little change in pressure; small decreases in volume are replenished from capacitance vessels [178]. Activation of veno-atrial stretch receptors by inflation of small balloons activates afferents and results in a reflex tachycardia (equivalent to the Bainbridge reflex) [179]. Similar results can be obtained by modest changes in blood volume, which alters veno-atrial stretch receptor activity without substantially changing blood pressure. For example, removal of 2mls of blood from adult rats (~10% blood volume, by arterial cannula) has no effect on mean blood pressure, but does increase *c-fos* expression in PVN spinally-projecting SPANs [180]. Similar results are seen in the rat water deprivation model of Toney and colleagues [181] and cardiovascular responses to water deprivation are reduced by GABA inhibition of the PVN [182]. Combined, these data strongly support the hypothesis that spinally-projecting SPANs are involved with the control of blood volume. 

### 5.3. Circadian Rhythm

Human blood pressure typically follows a distinct circadian rhythm, dipping overnight and rising in the morning [183]. It is notable that this rise occurs before waking up [184]. In some cases, hypertension is characterised by not just elevated blood pressure, but by a disturbed circadian pattern of blood pressure. Specifically, the usual night time blood pressure decrease fails to occur [185, 186]. This disturbance to the normal circadian cardiovascular pattern may contribute to the statistically higher number of cardiovascular deaths occurring first thing in the morning [187, 188]. Circadian rhythm is largely controlled at the level of the hypothalamus and, in particular, the suprachiasmatic nucleus (SCN) [189], which expresses a number of clock gene products [190, 191]. There are strong excitatory and inhibitory pathways from the SCN to the PVN of rats [99, 192], and, specifically, to spinally-projecting SPANs [31]. It is therefore possible that spinally-projecting SPANs play a role in circadian control of blood pressure.

### 5.4. Penile Erection 

The PVN is involved with penile erection in rats [193]. Oxytocinergic PVN neurones, which have a positive effect on penile erection, project to the hippocampus, the medulla and, most importantly, the spinal cord (For reviews see [194, 195, 196]). A retrograde tracing study using pseudorabies virus has shown central nervous system (CNS) innervations of the penis to include sympathetic pre-ganglionic neurones and the PVN, specifically parvocellular neurones in the PVN [197]. From here, it appears likely that continuation of the sympathetic pathway involves serotonin and/or dopaminergic neurotransmission in the lower spinal cord [198, 199].

### 5.5. Glucose Control 

Glucose-sensing by the brain requires input from a number of sensors, including the liver, carotid body and small intestine, as well as glucose-sensitive neurones in the brain [reviewed by 200]. Direct stimulation of the PVN by microinjection of glucose into the PVN causes an increase in sympathetic stimulation to brown adipose tissue (BAT) [201]. More indirect stimulation by peripheral glucose injection also activates the PVN, as shown by increased *c-fos* expression, particularly in identified SPANs [202, 203]. In brain slice experiments, SPANs are modulated by hypoglycaemia [44], but the mechanisms of this still need to be determined. Blood glucose levels are heavily regulated by the liver, which is under a degree of control by the autonomic nervous system. This includes sympathetic innervations projecting from the PVN [82, 84, 136, 204], which have been shown to alter blood glucose levels by influencing the liver [83, 137]. 

### 5.6. Regulation of Body Fat

Two papers have shown that PVN neurones project to brown adipose tissue (BAT) *via *spinally-projecting sympathetic nerves [205, 206]. In both cases, pseudorabies virus was injected into interscapular brown adipose tissue and, after a number of days, immunostaining for the virus could be found in the IML and the PVN. Although this demonstrates a connection from the PVN to BAT, the mechanism remains unclear. Pharmacological stimulation of the PVN by microinjection of stimulatory amino acids has been shown to produce differing effects. Amir [207] showed an increase in sympathetic outflow to BAT, Yoshimatsu and colleagues [208] found small decreases in sympathetic activity when the anorexigenic peptide cholecystokinin (CCK) was injected into the PVN, but showed a mainly a stimulatory response to injected glutamate [209]. Conversely, Madden and Morrison [210] showed an inhibitory effect. Obviously there is a potential involvement of SPANs with regulation of body fat, but the details remain unclear.

### 5.7. Thermoregulation

Thermosensitivity of neurones in the PVN was first shown by Inenaga *et al.* [211]. Poly-synaptic viral tracing studies in rats show that PVN SPANs are directly involved in thermoregulation [212]. Since then the region has been shown to be critical in thermoregulation [213-214]. Projections from the PVN to the RVLM, which contains sympathetic pre-motor neurones, are activated following heat exposure [215]. In addition, heat exposure activates spinally-projecting PVN neurones [216]. Whether this involves a simple vasomotor responses to elevated temperature (e.g., sympathetic vasodilation of skin in humans [217] or tail in the case of rodents [212]) or some other process is unknown. The PVN also has an effect on thermogenesis, *via *innervations of brown adipose tissue (see above).

### 5.8. Involvement with Heart Failure

Whilst chronic heart failure could not be described as a function of SPANs, the relationship between NO, GABA and SPAN activity is fundamental to the development of elevated sympathetic activity seen in human patients and animal models of heart failure [15, 33-34, 218]. NO is now well established as a neuromediator in the CNS where it is synthesised by neuronal nitric oxide synthase (nNOS) [reviewed by 219, 220]. nNOS is abundantly and constitutively expressed throughout the brain, but particularly in cells of the PVN; this includes expression by pre-autonomic neurones projecting both to the IML [221] and the RVLM [222, 223]. NO appears to increase the tonic inhibition of SPANs [224] by facilitating pre-synaptic release of GABA [223]. In models where chronic heart failure has been experimentally induced in rats, there are decreased levels of nNOS expression in the PVN [225], together with evidence of reduced neuronal activity [218]. Furthermore, the degree of tonic inhibition of rSNA by GABA (albeit assessed with bicuculline) was reduced in the chronic heart failure animals [35]. When combined these data strongly suggest that the tonic inhibition of sympathetic activity is maintained by an NO-dependent local release of GABA, and that chronic heart failure leads to a loss of NO activity, and thus to a loss of inhibitory control of sympathetic neurones [36] (Fig. **7**). It has recently been shown that AT_1_ receptor mRNA expression is increased in the PVN and, consequently, intra-PVN injection of AGII is more effective at increasing rSNA in the rat chronic heart failure model [226]. Further experiments are required to determine if the increase in angiotensin activity is causal, coincident or consequent to the decreased nNOS expression in the PVN.

## 6. CONCLUSIONS

Several studies have investigated the function and pharmacology of spinally-projecting SPANs in the PVN but, interestingly, we still know a great deal more about the expression of their neurotransmitters than anything else. There is relatively sparse information concerning which receptors are expressed by SPANs; GABA_A_, glutamate, adenosine A_1_ and NK1 receptors are all strong candidates, and SPANs can be indirectly modulated by NO and AGII. In terms of the modulation of SPANs, tonic inhibition is a recurrent theme and clearly central to their regulation. For example, NO, AGII and SP all appear to act by modulating this GABA tone. These neurones modulate the cardiovascular system, and it seems likely that they have other functions too. So a big question seems to be: what is the primary role of these neurones in cardiovascular function? There is evidence they become involved in the pathological increase in sympathetic activity associated with chronic heart failure, but this still leaves the function in normal, healthy animals unknown. Putative physiological functions include: volume regulation, glucose control, circadian rhythm and response to stress. Perhaps PVN SPANs play a role in all of these. The final question would then be, do the same neurones play a role in each of these functions, or does a mixed population of SPANs exist, each with a different receptor and neurotransmitter profile and each with a separate function? A great many further experiments will be required to finally answer these fascinating questions.

## ABBREVIATIONS

3V: = 3^rd^ ventricle
AGII: = Angiotensin II
AMPA: = α-amino-3-hydroxy-5-methyl-4-isoxazolepropionic acid
AP-5: = Amino-5-phosphonovaleric acid
AT_1_: = Angiotensin II type 1 receptor
AT_2_: = Angiotensin II type 2 receptor
BAT: = Brown adipose tissue
CCK: = Cholecystokinin
CNS: = Central nervous system
CVS: = Cardiovascular system
DMH: = Dorsomedial hypothalamus
FITC: = Fluorescein isothiocyanate
GABA: = γ-aminobutyric acid
HPA: = Hypothalamic-pituitary-adrenal
IML: = Intermediolateralis
NBQX: = 2,3-dihydroxy-6-nitro-7-sulfamoyl-benzo[f]quinoxaline-2,3-dione
NKA: = Neurokinin A
NKB: = Neurokinin B
NMDA: = N-methyl D-aspartate
nNOS: = Neuronal nitric oxide synthase
NO: = Nitric oxide
NTS: = Nucleus of the solitary tract
OB-R: = Leptin receptor
OT-R: = Oxytocin receptor
PACAP: = Pituitary adenylcyclase activating peptide
PKC: = Protein kinase C
rSNA: = Renal sympathetic nerve activity
RT-PCR: = Reverse transcriptase PCR
RVLM: = Rostral ventrolateral medulla
SCG: = Superiocervical ganglion
SCN: = Suprachiasmatic nucleus
SG: = Stellate ganglion
SP: = substance P
SPAN: = Sympathetic pre-autonomic neurone
T3: = Thyroid hormone
THDOC: = Tetrahydrodeoxycorticosterone
TSH: = Thyroid stimulating hormone
TR: = Thyroid hormone receptor
V_1a_-receptor: = Vasopressin 1a receptor
VIP: = Vasoctive intestinal peptide
VPAC_2_: = Vasoctive intestinal peptide/pituitary adenylcyclase activating peptide receptors type 2
